# Study protocol for a randomized controlled trial to improve the quality of life of housewives with musculoskeletal disorders: a health promotion intervention based on a participatory ergonomic approach—the Housewives Ergonomic Intervention (HEI) trial

**DOI:** 10.1186/s13063-021-05436-w

**Published:** 2021-07-26

**Authors:** Samaneh Norouzi, Sedigheh Sadat Tavafian, Rosanna Cousins, Hamidreza Mokarami

**Affiliations:** 1 Department of Health Education and Health Promotion, Tarbiat Modares University, Tehran, Iran; 2 Department of Psychology, Liverpool Hope University, Liverpool, UK; 3 Department of Ergonomics, School of Health, Shiraz University of Medical Sciences, Shiraz, Iran

**Keywords:** Health promotional intervention, Ergonomics, Quality of life, Housewives, Musculoskeletal disorders, Work-related stress, Risk factors

## Abstract

**Background:**

A variety of household chores expose women to a variety of biomechanical and psychosocial risk factors. A result of this is many housewives with musculoskeletal disorders. Given the interactive effects of these risk factors, it is necessary to consider multiple strategies to mitigate their effects. Accordingly, the present study will investigate the impact of a health promotion training program based on a participatory ergonomic approach towards a reduction in the prevalence of musculoskeletal disorders and an improvement in the quality of life of housewives.

**Methods:**

Iranian housewives aged 20–65 years currently attending a specialist health clinic due to a painful musculoskeletal complaint will be invited to join the study. Recruitment will continue until a sample of 160 women provides informed consent to participate. The study will be conducted using a mixed-methods protocol in two phases. In the first phase, psychosocial and biomechanical risk factors will be identified using a qualitative approach. In the next phase, the results from the qualitative approach will be used to develop a conceptual framework based on health promotion theories and an intervention program based on a participatory ergonomic approach designed. Participants will be randomly allocated into one of four groups: (1) biomechanical intervention group, (2) psychosocial intervention group, (3) multidisciplinary intervention group (both biomechanical and psychosocial intervention), and (4) a control group. Data will be collected using Rapid Entire Body Assessment (REBA), Visual Analog Scale (VAS), Work Ability Score (WAS), Hospital Anxiety and Depression Scale (HADS), and the 36-item Short-Form health survey (SF-36) at baseline in 3-month and 6-month follow-up assessments. The impact of the three interventions on musculoskeletal disorders, work ability, stress, and quality of life will then be evaluated.

**Discussion:**

The study will provide a practical approach to reducing stress, reducing musculoskeletal disorders, enhancing the ability to work, and improving the quality of life of women with musculoskeletal disorders associated with housework. If the designed interventions in the present study are effective, they will have the great practical potential for generalization to all housewives.

**Trial registration:**

ClinicalTrials.gov IRCT20200602047640N. Registered on 07 September 2020 with the IRCTID.

## Background

Many people have to engage in physical work to meet their personal and social needs. Whilst this work may promote their health and social development across both social and economic fields, it may also involve encountering various risk factors which, in turn, can lead to health problems [1, 2]. Work-related musculoskeletal disorders (WMSDs) are one of the most important of these health problems. These disorders are very common in both industrialized and developing countries, with statistics showing that nearly 150 million people worldwide are affected [3]. WMSDs cause pain and may lead to inability to perform physical activities, decreased functional capacity, weakness, and loss of individual independence. A consequence of all of these impairments is low health-related quality of life (HRQOL) [4].

WMSDs are multifactorial phenomena, and multiple biomechanical and psychosocial risk factors can contribute to their occurrence [5–7]. Similarly, the presence of various risk factors in work environments has indicated potentials for an interactive effect which can aggravate the impact of these risk factors on the occurrence of WMSDs [8, 9]. Therefore, it can be appreciated that intervention programs and measures that focus on the simultaneous control of these risk factors will be more effective in preventing these disorders than interventions that address these risk factors separately [10].

Housework is one of the most pressing jobs for women, and the risk of WMSDs is very high due to the presence of multiple risk factors associated with it [11–13]. Housework by itself may be a risk factor for WMSDs among women, and most importantly, these disorders limit women’s ability to protect themselves from its effects. Housework in unfavorable conditions, as well as potentials for stress caused by a high workload, can lead to more musculoskeletal problems and interfere with the healing process [14]. Studies have shown that housekeeping activities require twice as much energy as many other jobs and that various tasks routinely performed by a housewife could lead to stress and WMSDs [15].

The prevalence of musculoskeletal disorders in housewives is reported as 53% in Iran [16, 17], 49% in India [18, 19], and 84% in Bangladesh [20]. A review study by Habib and colleagues in Lebanon found that the biomechanical activities undertaken in the home increases the incidence of back, neck, and shoulder pain in housewives [4]. This being so, implementing preventive intervention programs for housewives is very important.

Women’s housework typically includes cooking, cleaning, washing, shopping, and caring for family members and children, all of which requires considerable time and involves physical, emotional, and mental activities. Altogether, these impose a high biomechanical and psychological burden on them [21, 22]. Similarly, a relatively recent study by Tavafian et al. showed that Iranian women do heavy household chores in poor physical conditions and in unfavorable psychosocial situations, which exacerbated their participant’s low back pain [23].

Despite numerous WMSD disorders in Iranian housewives, our review found that there has been no research-based intervention to reduce or control the biomechanical and psychosocial risk factors associated with WMSDs in Iranian housewives. It is the case that most studies have focused on formal work environments. In addition, few studies have examined the simultaneous effects of biomechanical and psychosocial interventions on reducing musculoskeletal disorders. Moreover, according to our review, there have been no special intervention studies for housewives in other countries either. Nevertheless, housework activities, due to their high physical and mental burden, expose housewives to WMSDs, especially in the lower back, neck, and shoulders [23, 24]. Therefore, biomechanical and psychosocial burdens of housework activities should be studied comprehensively from an ergonomics perspective, and appropriate risk reduction-based interventional programs and measures should be implemented to control or reduce these risk factors. Accordingly, using a qualitative approach, the present study will attempt to identify biomechanical and psychosocial risk factors related to WMSDs among Iranian housewives. Then, a health promotion intervention program will be implemented based on a participatory ergonomic approach towards improving the HRQOL of a sample of housewives with WMSDS symptoms, and the effectiveness of the program will be examined.

Based on the literature, it is presumed that this training interventional program will improve the HRQOL and work ability of housewives, and also that the severity of WMSDs and stress will be improved in the intervention groups relative to the control group. We also hypothesize that the use of a mixed biomechanical and psychosocial risk reduction interventional program will have an additive effective and this program will be more effective than each of the two separate intervention programs to reduce WMSDs and stress and improve the HRQOL and work ability consequences.

## Methods

### Aim, design, and outcomes

The aim of this study is to provide a practical approach to reducing musculoskeletal disorders, reducing stress, enhancing the ability to work, and improving the quality of life of women with musculoskeletal disorders associated with housework. The study will use an exploratory sequential mixed-methods design.

### Primary outcomes

- The selection/development of a health education and health promotion model based on the results of an initial qualitative study
- The design of health promotional interventions based on the results of previous steps (qualitative step)

### Secondary outcomes

- The effect of our educational intervention on the quality of life, ability to work, stress, and musculoskeletal problems.

Accordingly, the main research questions addressed in this study are as follows:

- Which health education and health promotion model will be preferred for use among the housewives?
- We are testing three interventions: which intervention is the most effective for improving quality of life and work ability, and reducing WMSD symptoms and stress?

### Ethical approval

The study protocol has been approved by the Medical Ethics Research Center of Tarbiat Modares University (reference: IR.MODARES.REC.1398.038). All participants will be provided with full information of their part in the study and assured that their information will be kept strictly confidential. All participants will be asked to complete a written informed consent form. This will provide a clear understanding that their participation is entirely voluntary, and they have a right to withdraw at any time during the study.

### Participants

The research population will comprise full-time housewives aged 20–65 years with a diagnosed MSD disorder. To meet these inclusion criteria, recruitment will be from women currently attending a specialist health clinic in Iran due to a painful musculoskeletal complaint. Invitations to join the study will continue to be offered to every eligible woman until we have achieved a sample of 160 housewives who provide informed consent to participate.

Full details of inclusion and exclusion criteria are provided in Table 1.

**Table 1:** Inclusion and exclusion criteria

**Inclusion criteria**	
Working-aged woman aged 20–65 years	
Diagnosed MSD and an MSD pain intensity score of 4 and higher based on the visual analog scale (VAS) [25, 26].	
The motivation and willingness to fully participate with any randomly allocated intervention	
Being married and living with the family	
Having no job other than housekeeping	
Not pregnant, nor breastfeeding	
**Exclusion criteria**	
Not able to actively attend the training sessions	
Diagnosis of a congenital disease that can affect skeletal tissue	
Having a child under 2 years of age	
Becoming pregnant during the study	
The occurrence of an event or accident that does not allow the person to complete the whole study procedure	
Taking medication to treat a mental illness	

### Study design

This exploratory sequential mixed-methods study will be conducted in two phases, each of which is described below. The schedule of enrolment, interviews, intervention, and assessment is shown in Table 2. This protocol was developed and reported according to the recommendations of the Standard Protocol Items: Recommendations for Interventional Trials (SPIRIT), and the clinical trial will be conducted and reported following the Consolidated Standards of Reporting Trials (CONSORT).

**Table 2:**
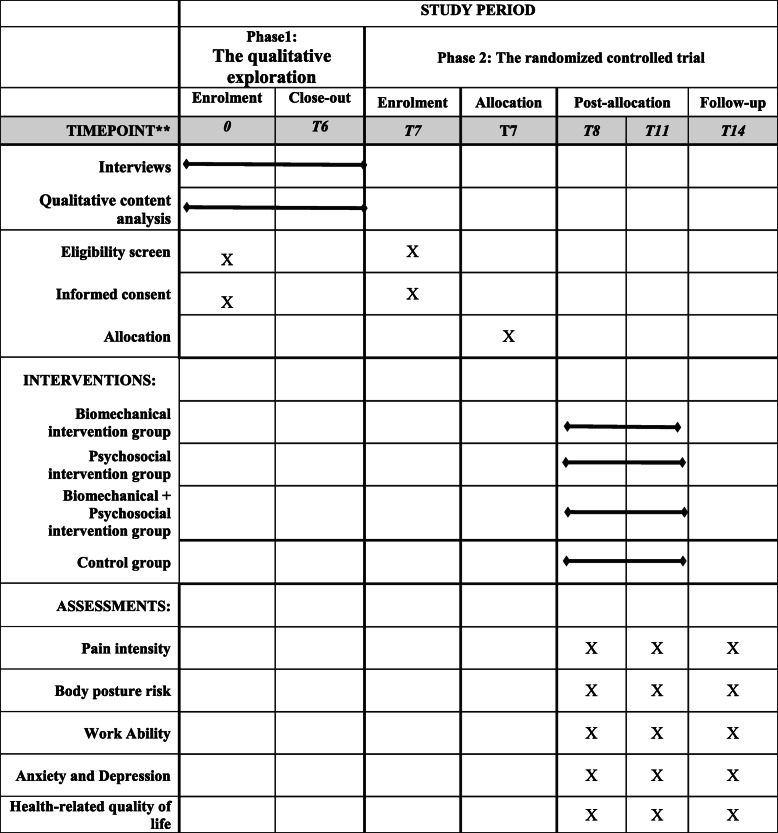
Schedule of enrolment, interviews, intervention, and assessment of the Housewives Ergonomic Intervention (HEI) trial, following the Standard Protocol Items Recommended for Clinical Trials (SPIRIT) guidelines

### Phase 1: The qualitative exploration

The qualitative study will be completed over 6 months. Semi-structured interviews with open-ended questions, and conventional qualitative content analysis methods will be conducted to investigate the biomechanical and psychosocial risk factors affecting WMSDs among the participants [27]. The interviews will take place face-to-face in a mutually convenient quiet environment without the presence of others. An interview guide has been prepared in line with the aim and objectives of the study. The initial question is a very general open-ended question around the participant’s work in the house, and they will be requested to provide a detailed answer [25]. Then further probing questions are asked according to this answer. The goal is to achieve a full understanding of the ergonomic and psychosocial circumstances of each participant’s WMSDs.

After completing each interview, it will be transcribed in full. The transcript will be then sent to each participant with a summary of key topics in each interview to ensure that the researcher has accurately interpreted the participants’ statements (member checking) [26], and if there are any ambiguities and inconsistencies, they will be resolved.

In addition, in parallel, we will use the Job Safety Analysis (JSA) by observation [28] to identify the most common musculoskeletal risk factors and harmful behaviors affecting the musculoskeletal health of each of the women in the study, to get an insight into their inability to protect their musculoskeletal health in their daily life. Finally, using the two approaches mentioned above (interview and observation), a health promotion model that best fits the qualitative study results will be developed.

### Phase 2: The randomized controlled trial

**Fig. 1:**
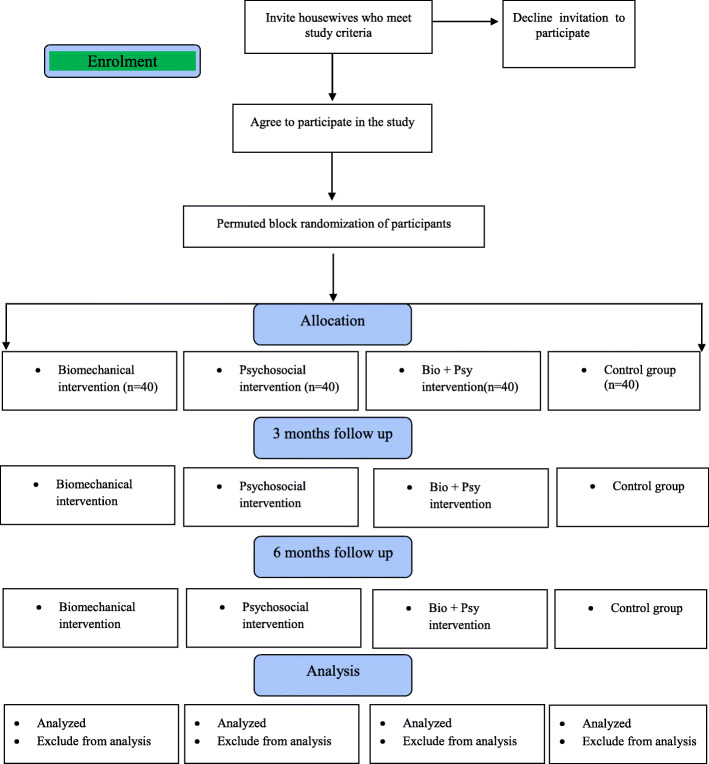
The flow chart of the randomized controlled protocol

Randomized controlled trials (RCTs) represent the most powerful way to evaluate public health interventions. Randomized controlled trials minimize the impact of confounding bias as the assignment of each of the study participants to an intervention group or the control group will be done solely by chance [29]. The flow chart of the randomized controlled protocol is shown in Fig. 1.

### The intervention programs

The conceptual framework of the health promotion model will be developed based on phase 1 findings from the qualitative study. Recruitment of women who meet the inclusion criteria will continue until the required sample size is achieved. At this point, all participants will be coded and blindly allocated into one of four intervention groups by the researchers using a permuted block randomization program: (1) biomechanical, (2) psychosocial, (3) multidisciplinary (biomechanical and psychosocial), or (4) control. Based on the design of the randomized controlled trial intervention, the impact of the different intervention programs on musculoskeletal disorders, work ability, and quality of life of the housewives will be evaluated. As shown in Fig. 1, measures will be collected at baseline, after 3 months, and after 6 months of following their respective program.

To implement the content of the three intervention programs, a participatory ergonomic approach will be used. The effectiveness of this approach in ergonomic interventions depends on the ability and active cooperation of participants [29]. The required training content for participants in the psychosocial intervention will be provided following the conceptual framework developed in the qualitative study and the resulting theory. Similarly, for participants in the biomechanical intervention group, the required training will be provided following the conceptual framework derived from the qualitative study and job safety analysis (JSA) method. Finally, the multidisciplinary intervention group will receive the training provided to the psychosocial and biomechanical groups: that is, they will do both interventions. The multidisciplinary groups will receive both of the other two interventions in full.

After providing the required training programs, the 40 participants in each of the three intervention groups will be divided into 4 subgroups of ten participants. There will be an expert, as a facilitator, who will each manage one of the 12 subgroups, overseen by a coordinator. Subgroup meetings will be held weekly to retrain the materials taught to the group members and discuss the actions taken by the members of the subgroups. The facilitators will be responsible for reviewing the training content provided to the participants in their subgroup, discussing the experiences of and the actions taken by the members of their subgroups, and performing the required assessment during the intervention period. The facilitators of the intervention groups will communicate with each other by forming a social group through virtual networks and face-to-face meetings and share the experiences of their subgroups with each other. The facilitator coordinator will ensure all aspects of the subgroup training and associated interactions are appropriate and intervene if necessary, to reduce any foreseeable risk of harm. The study group evaluators will remain blind to group allocation throughout the intervention, including knowledge of withdrawal from the study.

The facilitators will be provided with training for their role that will include information on how to manage work teams and how to use the risk assessment methods and other tools used in the present study. It will be made clear that the facilitators should not interfere in the participant’s training procedures or in the implementation of the interventions; their purpose is to play a facilitating role based on the principles of participatory ergonomics. In order to ensure the implementation of the interventions, reminder messages will sent to the participants on a weekly basis through virtual network. After the final evaluation and comparisons of the three intervention groups, the participants in the control group also will receive the most effective intervention, in accordance with ethical principles.

**Fig. 2:**
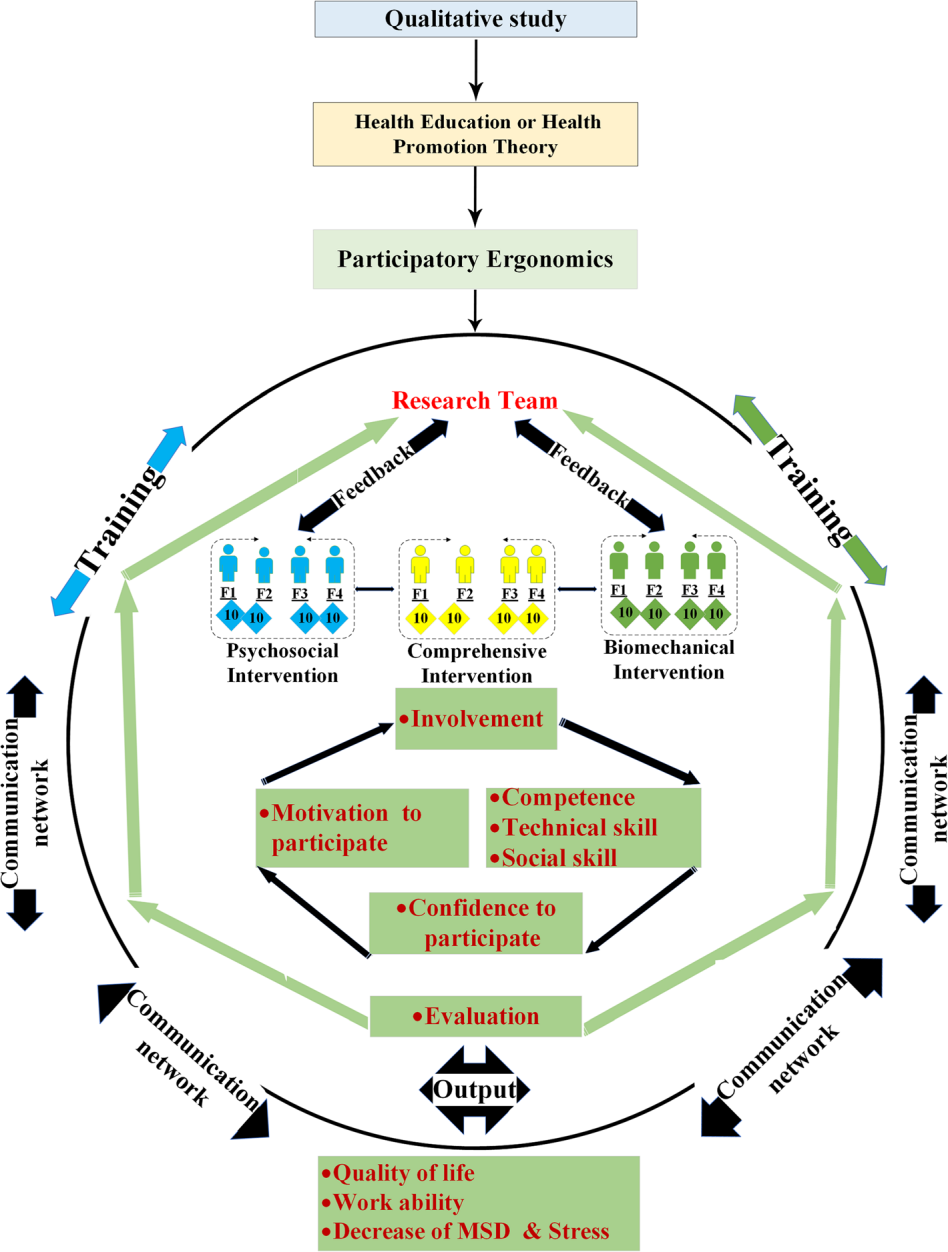
The program logic model for the participatory ergonomic approach (adapted from Haines & Wilson) [30, 31]

The program logic model developed based on the participatory ergonomic approach is presented in Fig. 2. The model provides an explanation of the study phases, the conceptual framework of participatory ergonomic approach and how it is implemented, the health promotion training program, and the process used to assess the expected outputs.

### Instruments

The instruments that will be used to collect the data are the Rapid Entire Body Assessment (REBA) [32], the Visual Analog Scale (VAS) [33], the Work Ability Score (WAS) [34], the Hospital Anxiety and Depression Scale (HADS) [35], and the 36-item Short-Form Health Survey (SF-36) [36]. Table 3 presents the general features of the mentioned instruments.

**Table 3:** Summary of the instruments used for data collection

Scale	Content	Scoring
VAS	Pain intensity	0 (no pain)–10 (severe pain)
REBA	The Rapid Entire Body Assessment	1 (no need for assessment), 11––15 (immediate assessment is required)
WAS	Work ability	0 (inability to perform activities)–10 (ability to perform activities)
HADS	The Hospital Anxiety and Depression Scale	0 (lowest anxiety/depression level)–21 (highest anxiety/depression level)
SF-36	Health-related quality of life	7 subscales: 0 (the worst)–100 (the best)

### Sample size and power calculations

Following the study conducted by Shariat [37], which similarly used a three-arm, parallel, RCT to investigate MSDs in office workers, the sample size we require in the quantitative phase to provide ample power was calculated as 30 persons for each group. This sample size was calculated to be sufficient at an alpha of 0.05 and a power of .80, to test for a difference between the groups. Nevertheless, sample size calculations are sensitive to error and complicated when drawing upon incomplete information in the literature [38]. Additionally, experience indicated we must consider a potential dropout rate of 30%. Hence, according to the following formula, we should work with an initial recruitment target of 40 participants in each group:

$$ n=\frac{{\left({Z}_{1-\frac{\alpha }{2}}+{Z}_{1-\beta}\right)}^2\left({\delta_1}^2+{\delta_2}^2\right)}{{\left(\overline{{\overline{X}}_1}-{\overline{X}}_2\right)}^2}=\frac{{\left(1.96+0.84\right)}^2\left({20.2}^2+{21.1}^2\right)}{{\left(-15\right)}^2}=29.9\cong 30 $$

N=30+30(0.3)≅40

In line with this approach, a total of 160 housewives aged 20–65 years with WMSDs will be recruited based on the health records available in the health centers of Akbarabad-Kavar city by taking into account the inclusion and exclusion criteria given in Table 1. Participants will be allocated into four groups (the three intervention groups plus one control group) based on permuted block randomization. The allocation is concealed by using opaque, sealed envelopes that are consecutively numbered and included each group’s name. Sampling using this randomization process gives each participant an equal chance of being placed in each group [39].

### Data analysis

#### Phase 1

To perform the process of qualitative content analysis, the audio file of each interview will be listened to attentively several times on the same day and it will be transcribed verbatim. To keep the data from the interviews confidential, a code will be assigned to each transcript. To come up with a general impression of the interviews and become fully immersed in the data, the audio files of the interviews and the transcripts will be reviewed several times, and any possible ambiguities and inconsistencies will be removed by comparing the audio files and the transcripts. The interviews will be audiotaped and a summary of the key issues in each interview will be then sent to each participant to ensure that the researcher will have accurately interpreted that participant’s comments (a “member check”) [26]. The process of data analysis will be performed continuously and simultaneously with the data collection process. All words, sentences, and paragraphs that are related in the analysis process will be considered as a single semantic unit. After merging the semantic units, the codes will be extracted. The codes together form the subcategories and then the main categories. Finally, upon the abstraction of the categories, the relevant themes will be identified. MAX.QDA software will be used to manage the data [40].

#### Phase 2

The collected data will be analyzed with SPSS software using descriptive statistics (including frequency, frequency percentage, mean, and standard deviation) and inferential statistics. We will undertake both “intention to treat” and “per protocol” approaches to the inferential statistical analyses to achieve fully understand the outcomes and manage bias in the face of any drop out. To compare the differences between the values obtained before the intervention and 3 and 6 months after the intervention in each group, generalized mixed models of analysis of variance for repeated measures will be used. We will also calculate differences between means of the independent groups with their respective 95% confidence intervals. All tests will be performed at a significance level of 0.05 (p <0.05). The Kolmogorov-Smirnov test will be used to test the normality of the data. The per protocol analyses will only include the participants who complete the intervention to which they were allocated.

## Discussion

This paper describes the HEI trial which will examine intervention programs to reduce harm from MSDs and associated stress seen in women as a result of housework. This will be the first comprehensive study to examine the impact of a participatory health promotion program based on a participatory ergonomic approach to reducing musculoskeletal disorders and improving the quality of life in housewives. The ultimate goal of the study is to improve the quality of life, increase work ability, and reduce stress and the severity of musculoskeletal pain in housewives. Housewives in Iran account for a high percentage of the population. Despite the high prevalence of skeletal disorders affecting this community, no interventions have been made to reduce their musculoskeletal injuries, despite the potential benefits to individuals, families, and society. Therefore, this study aims to identify factors underlying musculoskeletal disorders and implement a purposeful intervention program as an effective step to reduce these disorders and improve the quality of life of housewives in Iran.

This study has several robust design features detailed as follows:

1. *The use of a mixed-methods protocol to identify risk factors:* Housework refers to a series of routine activities done by a person during the day. Thus, housewives are exposed to multiple psychosocial and biomechanical risk factors. To the best of our knowledge, to date no study has been conducted to identify the multiple psychosocial and biomechanical risk factors that account for musculoskeletal disorders among housewives to design an educational intervention program for the target population. As such, psychosocial stressors will be identified in this study using in-depth and face-to-face interviews. Besides, using the JSA method, the living and work environments of housewives will be assessed, and the most important biomechanical risk factors will be identified. Accordingly, a training intervention program that is bespoke to the population of housewives under study will be implemented.
2. *The use of a multidisciplinary (biomechanical and psychosocial) method to implement health promotion training programs:* Based on the reviews that have been done by the research team, the few studies that have been conducted to reduce musculoskeletal disorders in housewives have focused on only one of the known risk factors. However, we know there is a multiplicity of risk factors, so there is now a need to conduct a study using comprehensive interventions in this target group. Numerous studies have shown the interactive effects of biomechanical risk factors such as poor physical conditions and workload, and psychosocial risk factors such as lack of support from others have concluded that the interactive effect of risk factors underlying musculoskeletal disorders is broader than the effect of each risk factor alone. Thus, the present study will examine the gap in the literature to find out whether multidisciplinary interventions are more effective in reducing musculoskeletal disorders among housewives than biomechanical or psychosocial interventions performed separately. Accordingly, four separate groups will be evaluated in this study. This being so, we will be able to deduce which of these interventions is most effective in reducing musculoskeletal disorders.
3. *Performing a randomized controlled trial:* This study will be the first randomized controlled trial performed on housewives to evaluate the effectiveness of comprehensive and multifaceted interventions. Randomized controlled trial designs are held as the gold standard, with best internal validity and least distortion in clinical interventions and healthcare. However, they have rarely been used to evaluate the effectiveness of intervention programs in informal work environments such as the home and housewives’ communities. The evaluations of this study will show that these interventions are possible and feasible in work environments, including the home environment.
4. *The use of participatory ergonomics to implement the intervention program:* Using participatory ergonomics is another high-quality contribution of this study, as this technique engages end-users in the entire study process. According to the principles of this ergonomic approach, improving the health literacy level of housewives will increase their motivation and participation in intervention programs and afford more effective implementation. In addition, the use of network communications and facilitator training in the present study can significantly affect the effectiveness of interventions [30, 31].
5. *Follow-up period:* The gap in the majority of intervention studies is that they have focused on short-term results. The present study explores the effectiveness of the training intervention over a longer period: collecting data at three time points: before the intervention (baseline) and follow-up 3 and 6 months after the intervention.

We do recognize that this study will have some limitations in so far as we must limit the randomized controlled trial to housewives residing in one region of Iran. This, however, will enable us to maintain good control of the test procedure. Another limitation of this study is the need to use self-reporting tools, and thus, there is the possibility of memory error, lack of clarity, social concerns, and individual biases affecting the results. Nevertheless, to compensate for this necessity, valid standard tools will be used. Besides, it is anticipated that the level of participation in the study will remain high and atrophy in the sample size low, because the intervention will continuously offer a reward to the participants in improvement in WMSDs and quality of life. We anticipate this will be so for all the training groups, even if the time commitment for training differs according to group.

## Conclusion

This study will provide a practical approach to improving quality of life, reducing stress, reducing musculoskeletal disorders, and improving work ability among housewives. If the interventions are effective, the training protocol that will be developed in the present study will have great potentials for being implemented as an educational intervention among housewives and in other environments.

### Trial status

The study is ongoing. Recruitment opened in November 2020 and will continue until all housewives required for the trial are enrolled, planned to be in April 2021. The duration of the study period will be 1.5 years and will be finished in April 2022.

## Data Availability

Not applicable. The manuscript does not report data. The datasets subsequently generated and/or analyzed during the current study may be made publicly available following conclusion of ongoing research. Requests for data may be made at any time to the corresponding author.

## Abbreviations

WMSDs: Work-related musculoskeletal disorders
MSD: Musculoskeletal disorder
CONSORT: Consolidated Standards of Reporting Trials
MMR: Mixed methods research
RCT: Randomized controlled trial
VAS: Visual analog scale
REBA: Rapid Entire Body Assessment
WAS: Work Ability Score
HADS: Hospital Anxiety and Depression Scale
SF-36: Short Form Health Survey 36 Items Version 1.0
